# Electronic Dietary Intake Assessment (e-DIA): Comparison of a Mobile Phone Digital Entry App for Dietary Data Collection With 24-Hour Dietary Recalls

**DOI:** 10.2196/mhealth.4613

**Published:** 2015-10-27

**Authors:** Anna M Rangan, Sarah O'Connor, Valentina Giannelli, Megan LH Yap, Lie Ming Tang, Rajshri Roy, Jimmy Chun Yu Louie, Lana Hebden, Judy Kay, Margaret Allman-Farinelli

**Affiliations:** ^1^ School of Molecular Bioscience Charles Perkins Centre University of Sydney Camperdown Australia; ^2^ Human Centred Technology School of Information Technology University of Sydney Camperdown Australia

**Keywords:** validity, dietary assessment, mobile phone app, young adult

## Abstract

**Background:**

The electronic Dietary Intake Assessment (e-DIA), a digital entry food record mobile phone app, was developed to measure energy and nutrient intake prospectively. This can be used in monitoring population intakes or intervention studies in young adults.

**Objective:**

The objective was to assess the relative validity of e-DIA as a dietary assessment tool for energy and nutrient intakes using the 24-hour dietary recall as a reference method.

**Methods:**

University students aged 19 to 24 years recorded their food and drink intake on the e-DIA for five days consecutively and completed 24-hour dietary recalls on three random days during this 5-day study period. Mean differences in energy, macro-, and micronutrient intakes were evaluated between the methods using paired t tests or Wilcoxon signed-rank tests, and correlation coefficients were calculated on unadjusted, energy-adjusted, and deattenuated values. Bland-Altman plots and cross-classification into quartiles were used to assess agreement between the two methods.

**Results:**

Eighty participants completed the study (38% male). No significant differences were found between the two methods for mean intakes of energy or nutrients. Deattenuated correlation coefficients ranged from 0.55 to 0.79 (mean 0.68). Bland-Altman plots showed wide limits of agreement between the methods but without obvious bias. Cross-classification into same or adjacent quartiles ranged from 75% to 93% (mean 85%).

**Conclusions:**

The e-DIA shows potential as a dietary intake assessment tool at a group level with good ranking agreement for energy and all nutrients.

## Introduction

The collection of accurate dietary consumption data is important in the field of nutritional epidemiology in order to establish true relationships between nutrition and health status. The food record (weighed or estimated portions) is a traditional method used to record amounts and types of foods and beverages consumed prospectively, thus limiting recall bias [1,2]. However, one of the main limitations of food records is the high burden placed upon respondents to record this detailed dietary information [1,2]. For researchers, food record entries must be manually entered for analysis with food and nutrient software programs which takes significant time. Thus, improvements to methods for prospective dietary recording would be beneficial for research participants and researchers alike.

With 81% of Australians regularly using a mobile phone [3], the collection of dietary intake records using a mobile phone app has the potential to be more convenient for recording entries than conventional paper-based food records [4,5]. Mobile phone apps that use image-based food records rather than digital entry of foods are also increasingly available [6-9]. A recent review by our group concluded that mobile phone use to record dietary intake was preferred by users over conventional methods and offers the potential to reduce research costs through automated coding [6].

A number of commercial mobile phone apps such as MyFitnessPal and Lose It provide a platform for users to digitally record foods and beverages consumed and have these records integrated with food composition databases to calculate nutrients [10]. Only one, Easy Diet Diary, uses an Australian database of foods. However, the feedback display of nutrient intakes by these apps might elicit unintended behavior changes. We aimed to purposely design a mobile phone app (the electronic Dietary Intake Assessment, e-DIA) that would allow digital recording of all foods and beverages consumed, either weighed or estimated, but provide no nutrient content feedback. The aim of this study was to compare the energy and nutrient intakes collected with e-DIA against 24-hour dietary recalls and evaluate e-DIA’s potential as a dietary assessment tool in research.

## Methods

### Study Sample

**Figure 1:**
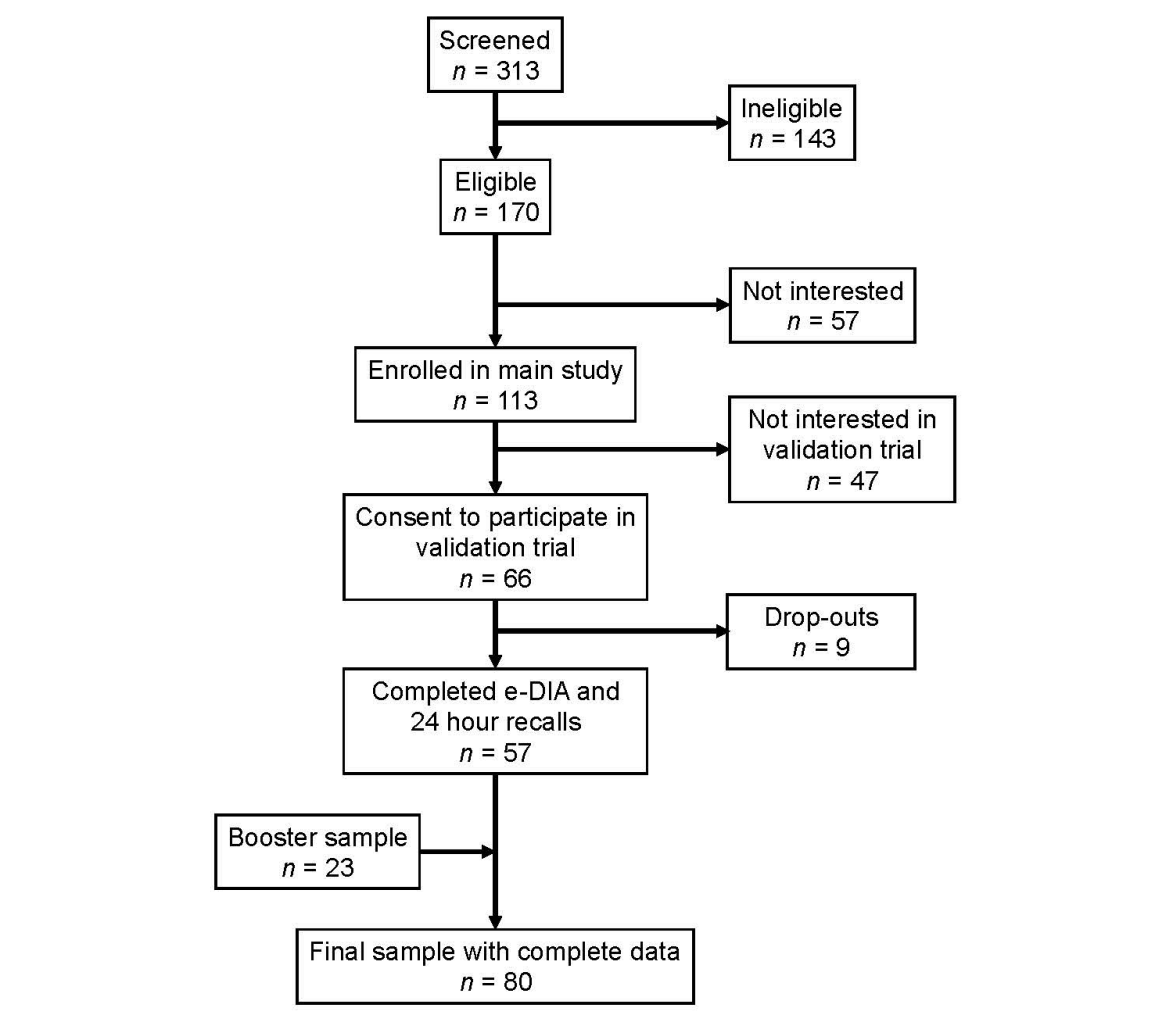
Flow chart of participant recruitment.

Students enrolled in a study aimed at assessing university students’ dietary intakes were invited to participate in this validation study. Recruitment methods for the larger study included email and poster advertisements on the university campus, which included a weblink to an online screening survey. Out of 313 students who completed the survey, 170 were eligible and 113 students were enrolled at an interview during which the study protocol was explained and written informed consent was obtained. From the enrolled students, 66 agreed to participate in the validation study and 57 completed both e-DIA and 24-hour dietary recalls from March to April 2014. To boost sample size, an additional 23 students were recruited in August 2014 by the same methods. This resulted in a final sample of 80 students (Figure 1). Inclusion criteria included being a full-time student aged 19 to 24 years, being enrolled in the second, third, or fourth year of study within the Science or Engineering departments, and owning a mobile phone. Nutrition and health science students were excluded. As an incentive to participate, all students were entered in a drawing to win an Apple iPad Mini after completion of the study. The study was conducted in agreement with the National Statement on Ethical Conduct in Human Research [11], and ethical approval was obtained from the university’s Human Research Ethics Committee (2014/136).

### e-DIA Mobile Phone App

**Figure 2:**
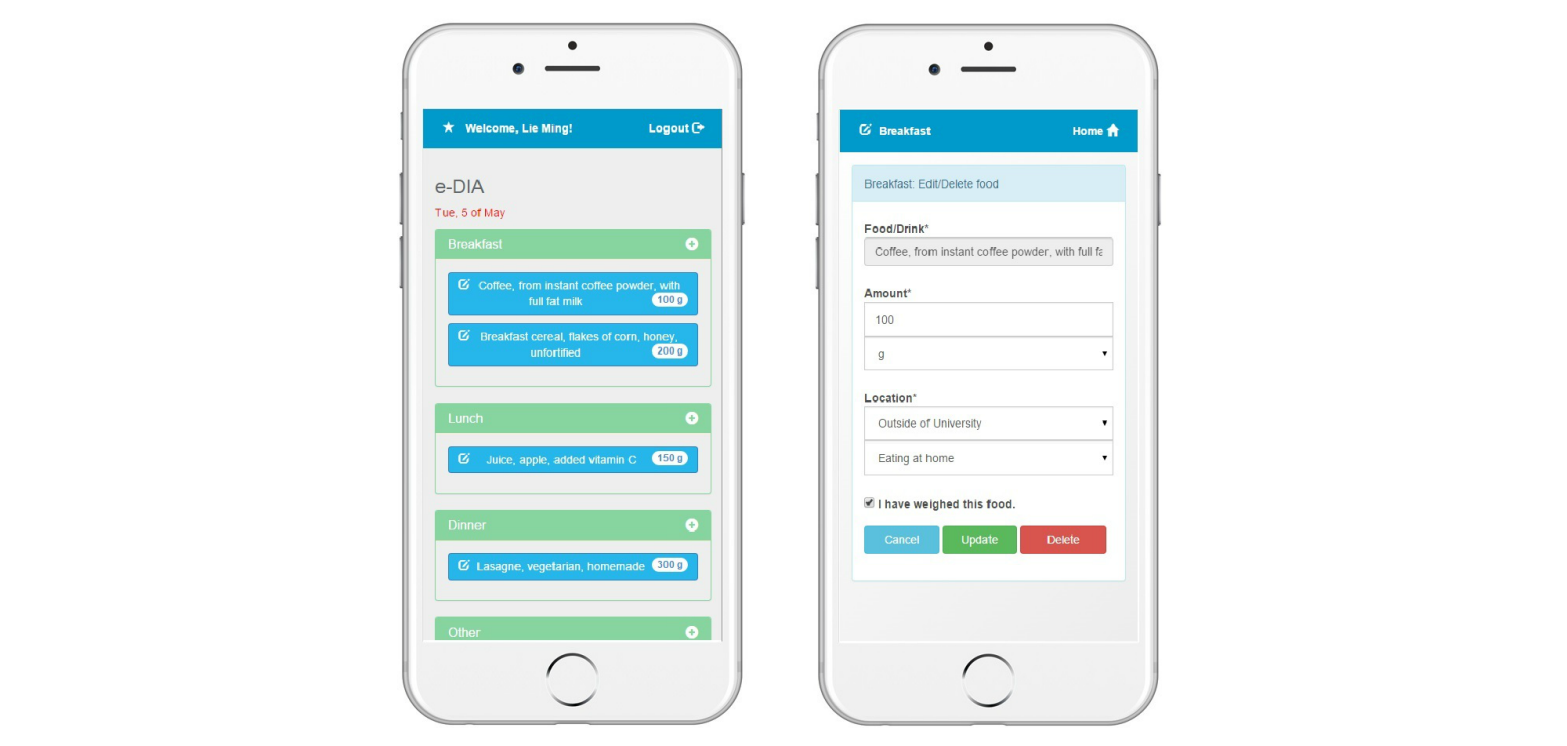
Screenshots of the electronic Dietary Intake Assessment (e-DIA) app.

Students downloaded the e-DIA app using an Android or iOS platform on their own mobile phone. To record intake, the user selects the meal occasion during which the food or beverage is consumed (breakfast, lunch, dinner, or other) which opens the Edit/Delete screen (Figure 2). On this screen the user selects the Food/Drink field to search for and choose the food or drink they consumed. A search-as-you-type function which begins to show a string of options once three letters are typed was built into the app, as was a favorites function for entry of foods commonly consumed by the participant. These additional navigation functions were added after usability testing of a previous prototype of e-DIA (results unpublished). The list of foods for this search function was based on the 2007 Australian Food, Supplement, and Nutrient Database (AUSNUT 2007)—the most recent food composition database at the time this research was conducted [12]. To log foods that were not listed or could not be found in the AUSNUT 2007 database, participants were asked to enter these manually into e-DIA. The amounts of foods and beverages consumed and location of consumption were also recorded (Figure 2). Data were uploaded to the research administrator’s website each day at midnight, after which the user could no longer access or view the record.

### Procedure

At an initial clinic appointment on the university campus, anthropometric data were collected by the study investigators. Height was measured to the nearest 0.5 cm, weight to the nearest 0.1 kg (without heavy clothing or shoes), and waist circumference to the nearest 0.5 cm, according to the Anthropometry Procedures Manual from the National Health and Nutrition Examination Survey (National Center for Health Statistics, US Department of Health and Human Services) [13].

Participants were instructed to complete five consecutive days of food records including three weekdays and two weekend days using e-DIA. Participants practiced selecting and entering food items and weights, and written instructions were included on how to choose foods from the database, how to enter mixed recipes, and how to estimate portion sizes when eating away from home. Participants were asked to weigh foods using the scales supplied (Salter 1066WHDR); an instruction booklet was provided. If participants were unable to weigh the foods, they were instructed to estimate portion sizes using metric cups and spoons supplied. Starting days were staggered so that all days of the week were represented across the sample. Participants were sent a text message reminder prior to each collection day which encouraged them to maintain their usual diet.

As a reference measure, three 24-hour dietary recalls were collected on three random days (including weekend days) during the five-day study period. Appropriate calling times were established at the convenience of the participants. The standard 24-hour dietary recall interview multi-pass script adapted from the Five-Step Multiple-Pass Method by the US Department of Agriculture [14] was used for the 30-minute telephone interviews, and participant responses were recorded on a standardized 24-hour dietary recall form. In addition to the metric cups and spoons, a food model booklet [15] was provided to aid in the estimation of food and beverage portion sizes for the 24-hour dietary recalls.

### Data Coding and Cleaning

All entries were checked the following day by study investigators, and participants were contacted to clarify manually entered food items and obvious inconsistencies such as gross data entry errors and skipped meals.

Data collected using the e-DIA mobile web app were stored in a cloud-based database, and records were linked to food items in the AUSNUT 2007. If the nutrient composition of manually entered food items was known, study investigators added the information to the database; if unknown, investigators coded to the closest match. Food intake data from the 24-hour dietary recalls were manually entered by trained study investigators into FoodWorks 7 Premium [16], a nutrient analysis software system using the AUSNUT 2007 database [12].

Energy and nutrient intakes from the 24-hour dietary recalls and e-DIA were examined for outliers and checked against the original 24-hour dietary recall for obvious errors in data entry. Errors made by the participant in the e-DIA were left unaltered, and no outliers were removed to provide a more accurate indication of the relative validity of the e-DIA method. Vitamin and mineral supplements were excluded from analysis.

### Statistical Analysis

Mean or median intakes of energy and nutrients from three days of 24-hour dietary recalls and five days of e-DIA were calculated and differences determined using paired *t* tests (normally distributed data including energy and macronutrients) or Wilcoxon signed-rank test (skewed data for alcohol and micronutrients). Correlations between the two methods were measured using Pearson product-moment correlation coefficients (or Spearman rank correlation coefficients for skewed data) for unadjusted, energy-adjusted and deattenuated data. Energy-adjusted nutrients were obtained by applying the residual method [2]. Deattenuated nutrient intakes corrected for within-person variation in both 24-hour dietary recalls and e-DIA were estimated using the Multiple Source Method [17]. Cross-classification and Bland-Altman plots [18] were used to assess the agreement between the 24-hour dietary recalls and e-DIA for energy and nutrients. Cross-classification examined the proportions of participants classified into the same, same or adjacent, or extreme quartiles of energy-adjusted intakes. Bland-Altman plots were presented to assess bias within the intake range. All data were analysed using SPSS Statistics version 22.0 (IBM Corp) [19] and a *P* value <.05 was considered statistically significant.

## Results

A sample of 80 students (30 male) completed five days of e-DIA and three days of 24-hour dietary recalls (Figure 1). The main reason given for not participating or dropping out of the study was due to time restraints and heavy workloads. Mean body mass index (BMI) was 22.6 kg/m^2^ (SD 3.8) with 63 participants (79%) in the healthy weight range (BMI 18.5-24.9), nine overweight (BMI 25.0-29.9), four obese (BMI>30.0), and three underweight (BMI<18.5). Mean waist circumference was 70 cm (SD 6.6) for females and 81 cm (SD 10.5) for males. One participant did not consent to disclosing her anthropometric data. The majority of participants lived at home with family (70%), with English being the most commonly spoken language at home (75%).

Mean and median intakes of energy and nutrients reported by 24-hour dietary recall and e-DIA are shown in Table 1. Differences between energy and nutrient intakes were mostly small, and none were statistically significant.

**Table 1:** Mean and median daily intakes of energy and nutrients measured by three days of 24-hour dietary recall (24HR) and five days of electronic Dietary Intake Assessment (e-DIA).

Energy and nutrients	e-DIA		24HR		Difference
	Mean (SD)	Median	Mean (SD)	Median	Mean (SD)
Energy, kJ	8148.2 (2495.2)	7699.1	8182.2 (2575.1)	7625.4	−34.3 (2090.3)
Protein, g	88.7 (33.5)	86.7	91.3 (35.0)	85.2	−2.5 (22.5)
Total fat, g	74.6 (25.6)	70.3	76.0 (31.4)	68.6	−1.4 (23.5)
SFA^a^, g	28.8 (11.8)	26.4	30.1 (16.4)	26.8	−1.3 (10.8)
MUFA^b^, g	28.4 (10.9)	26.5	28.7 (11.7)	25.8	−0.3 (9.8)
PUFA^c^, g	11.6 (4.4)	11.2	11.4 (4.6)	10.5	0.3 (4.5)
Carbohydrate, g	213.3 (82.6)	204.8	209.0 (67.5)	197.4	4.3 (70.1)
Sugars, g	80.1 (41.8)	72.9	88.1 (50.4)	78.3	−8.0 (43.7)
Starch, g	130.5 (57.1)	122.6	120.9 (46.9)	114.7	9.6 (44.6)
Fiber, g	22.0 (8.0)	21.5	21.5 (8.5)	20.0	0.5 (7.9)
Alcohol, g	4.8 (10.7)	0.1	3.9 (10.0)	0.0	0.9 (4.7)
Vitamin A RE^d^, μg	812.2 (961.5)	634.4	866.0 (1403.3)	653.1	−53.8 (574.1)
Thiamin, mg	1.5 (1.0)	1.3	1.5 (0.8)	1.3	0.1 (0.9)
Riboflavin, mg	1.9 (0.9)	1.9	2.1 (0.9)	1.9	−0.2 (0.6)
Niacin, mg	43.5 (18.5)	42.3	45.8 (21.6)	41.3	−2.3 (16.5)
Folate DFE^e^, μg	343.2 (212.4)	295.2	365.1 (232.8)	313.9	−21.9 (143.4)
Vitamin C, mg	90.7 (57.5)	76.9	106.8 (89.7)	88.4	−16.1 (78.1)
Vitamin E, mg	8.6 (4.5)	7.7	8.5 (3.9)	7.9	0.1 (3.8)
Calcium, mg	705.4 (318.0)	686.2	725.6 (317.7)	658.4	−22.0 (230.3)
Iron, mg	12.7 (9.3)	11.4	12.1 (5.6)	10.8	0.6 (7.5)
Zinc, mg	11.3 (7.1)	10.0	11.2 (4.6)	10.5	0.2 (6.7)
Magnesium, mg	333.1 (133.7)	311.4	323.4 (106.8)	312.4	10.0 (100.2)
Phosphorus, mg	1403.0 (511.6)	1388.1	1383.9 (470.8)	1324.2	18.6 (321.9)
Sodium, mg	2712.3 (1480.0)	2375.5	2561.5 (952.4)	2433.3	151.0 (1234.7)
Potassium, mg	2701.1 (1003.9)	2679.2	2732.1 (801.3)	2632.6	−31.3 (768.5)

^a^SFA: saturated fatty acids

^b^MUFA: monounsaturated fatty acids

^c^PUFA: polyunsaturated fatty acids

^d^RE: retinol equivalents

^e^DFE: dietary folate equivalents

Table 2 shows the correlation coefficients between the 24-hour dietary recalls and e-DIA. All correlation coefficients were statistically significant (*P*<.001). Correlations for unadjusted intakes were in the range 0.50 to 0.79 (mean correlation of 0.66 for all nutrients), energy-adjusted correlations were in the range 0.40 to 0.78 (mean 0.63), and deattenuated correlations were in the range 0.55 to 0.79 (mean 0.68). The highest correlations were found for protein and saturated fats while the lowest correlation was found for polyunsaturated fats. Deattenuated correlation coefficients were generally higher than unadjusted or energy-adjusted coefficients but differences were small.

Quartile cross-classification of nutrients with the 24-hour dietary recalls and e-DIA placed 75% to 93% (mean 85%) of the participants into the same or adjacent quartile, with the highest ranking agreement for fiber and the lowest for iron. Cross-classification into extreme quartiles ranged from 0% to 9% (mean 1%) with monounsaturated fatty acids (MUFA), thiamine, and iron having the greatest proportion of extreme misclassification.

**Table 2:** Correlation coefficients and cross-classification of energy and nutrients between three days of 24-hour dietary recall and five days of electronic Dietary Intake Assessment.

Energy and nutrients	Correlation coefficients^a,b^			Cross-classification into quartiles^c^		
	Unadjusted	Energy- adjusted	De- attenuated	Same	Same or adjacent	Extreme
Energy, kJ	0.66	—	0.68	38	81	0
Protein, g	0.79	0.77	0.79	58	87	1
Total fat, g	0.68	0.71	0.69	41	81	5
SFA^d^, g	0.75	0.78	0.76	46	91	0
MUFA^e^, g	0.62	0.62	0.64	45	79	6
PUFA^f^, g	0.50	0.43	0.55	46	82	2
Carbohydrate, g	0.64	0.75	0.67	49	87	2
Sugars, g	0.56	0.57	0.62	48	84	0
Starch, g	0.65	0.65	0.72	46	89	2
Fiber, g	0.54	0.63	0.64	59	93	1
Alcohol, g	0.77	0.69	0.62	44	88	1
Vitamin A RE^g^, μg	0.61	0.66	0.61	49	88	4
Thiamin, mg	0.61	0.40	0.66	35	79	9
Riboflavin, mg	0.77	0.70	0.76	45	90	0
Niacin, mg	0.69	0.58	0.71	53	83	2
Folate DFE^h^, μg	0.69	0.72	0.71	58	89	2
Vitamin C, mg	0.68	0.71	0.75	56	89	0
Vitamin E, mg	0.53	0.60	0.56	40	85	1
Calcium, mg	0.75	0.57	0.72	40	80	2
Iron, mg	0.57	0.42	0.61	34	75	6
Zinc, mg	0.69	0.54	0.70	49	82	2
Magnesium, mg	0.71	0.69	0.72	48	88	0
Phosphorus, mg	0.76	0.69	0.78	53	87	1
Sodium, mg	0.60	0.59	0.60	48	88	5
Potassium, mg	0.64	0.68	0.68	59	92	2

^a^Pearson correlation coefficients used for energy and macronutrients; Spearman rank correlation coefficients used for alcohol and micronutrients.

^b^All correlations were significant (*P*<.001).

^c^Based on energy-adjusted data.

^d^SFA: saturated fatty acids

^e^MUFA: monounsaturated fatty acids

^f^PUFA: polyunsaturated fatty acids

^g^RE: retinol equivalents

^h^DFE: dietary folate equivalents

**Figure 3:**
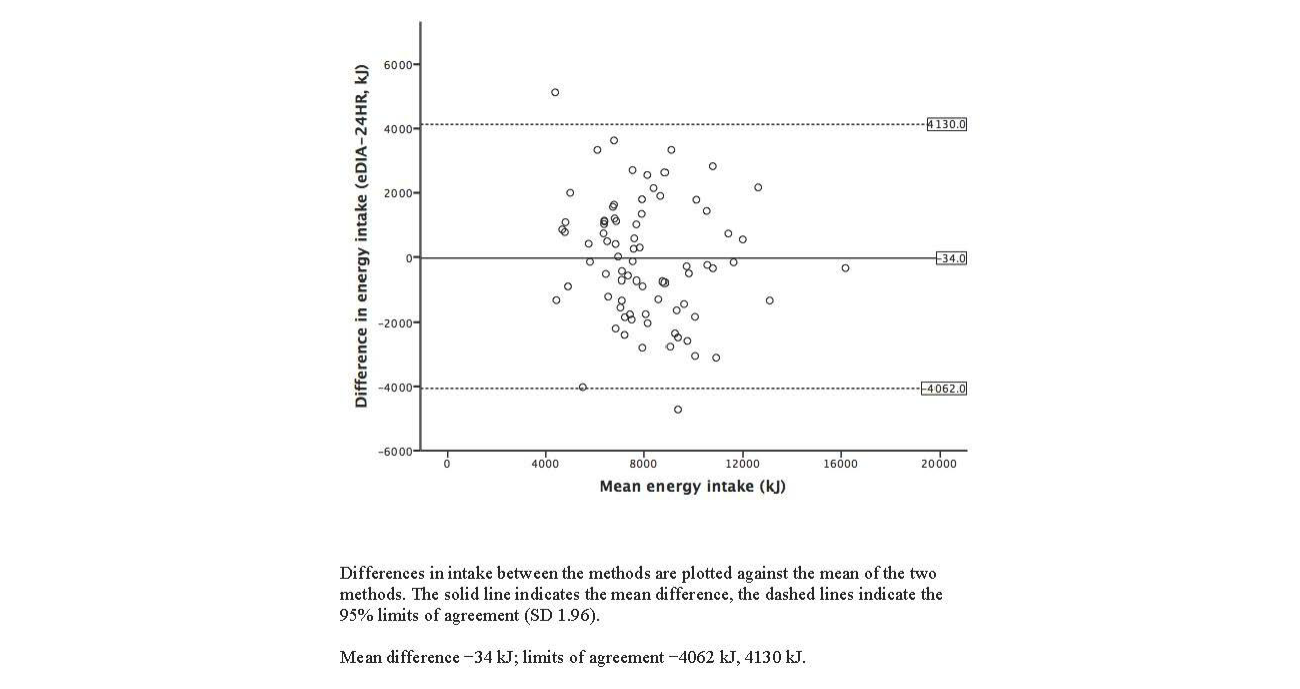
Bland-Altman plot of 24-hour dietary recalls (24HR) and electronic Dietary Intake Assessment (e-DIA) for energy intake.

**Figure 4:**
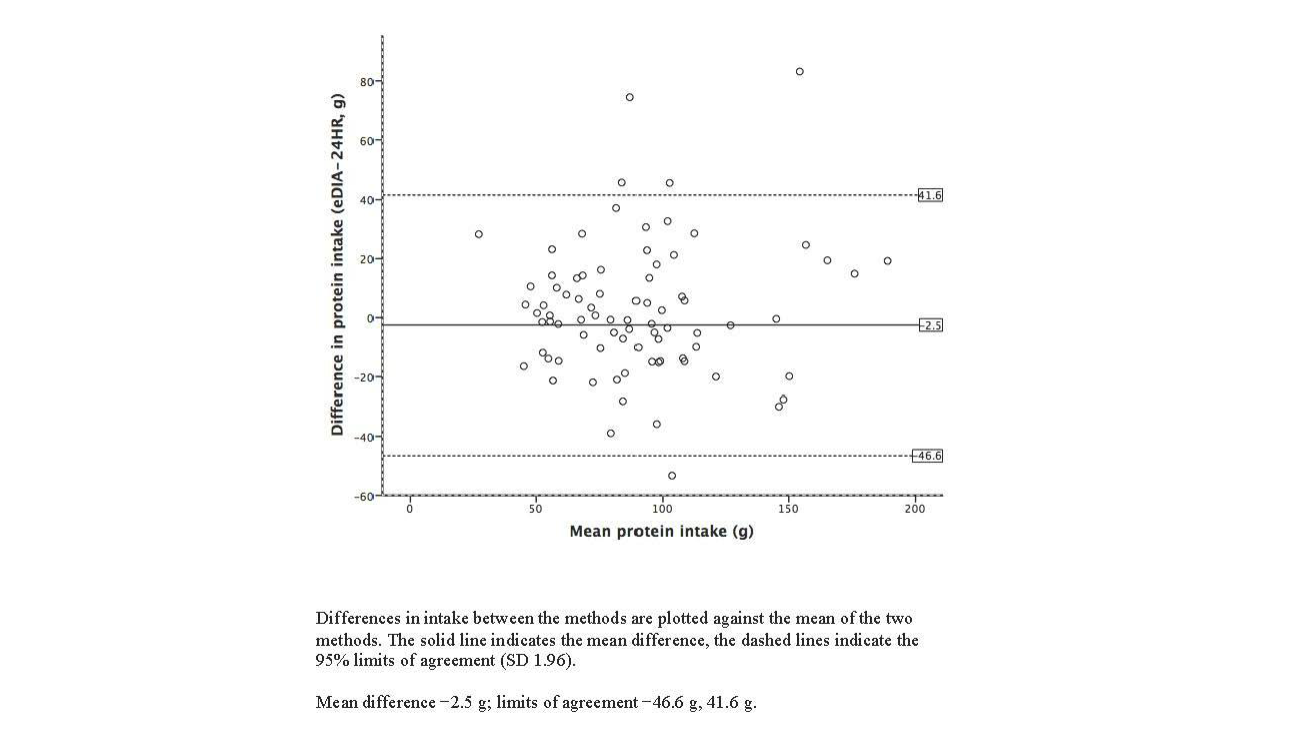
Bland-Altman plot of 24-hour dietary recalls (24HR) and electronic Dietary Intake Assessment (e-DIA) for protein intake.

**Figure 5:**
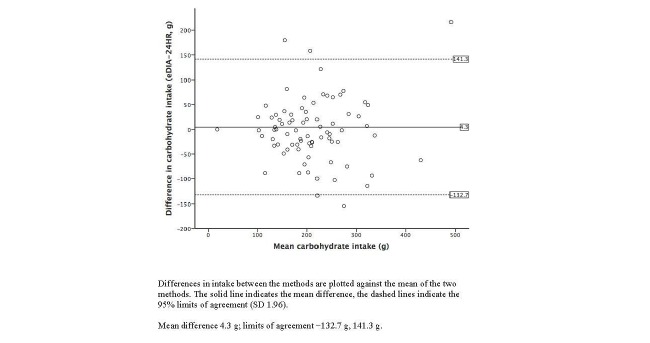
Bland-Altman plot of 24-hour dietary recalls (24HR) and electronic Dietary Intake Assessment (e-DIA) for carbohydrate intake.

**Figure 6:**
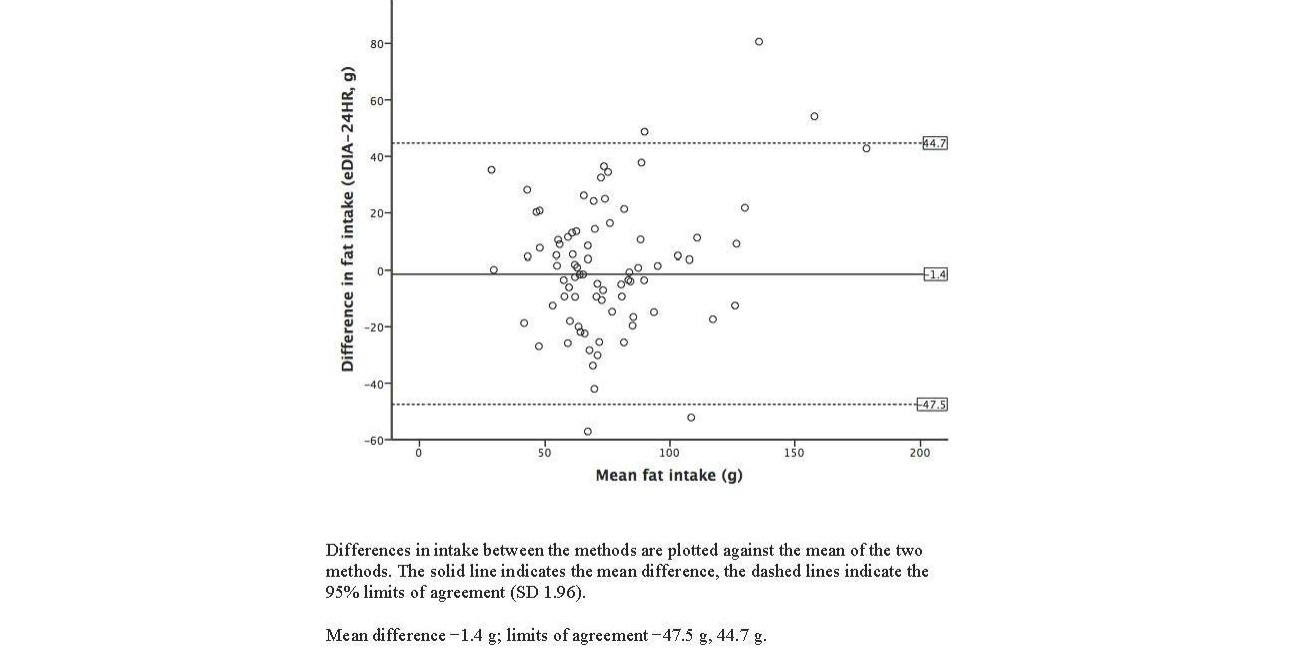
Bland-Altman plot of 24-hour dietary recalls (24HR) and electronic Dietary Intake Assessment (e-DIA) for fat intake.

**Figure 7:**
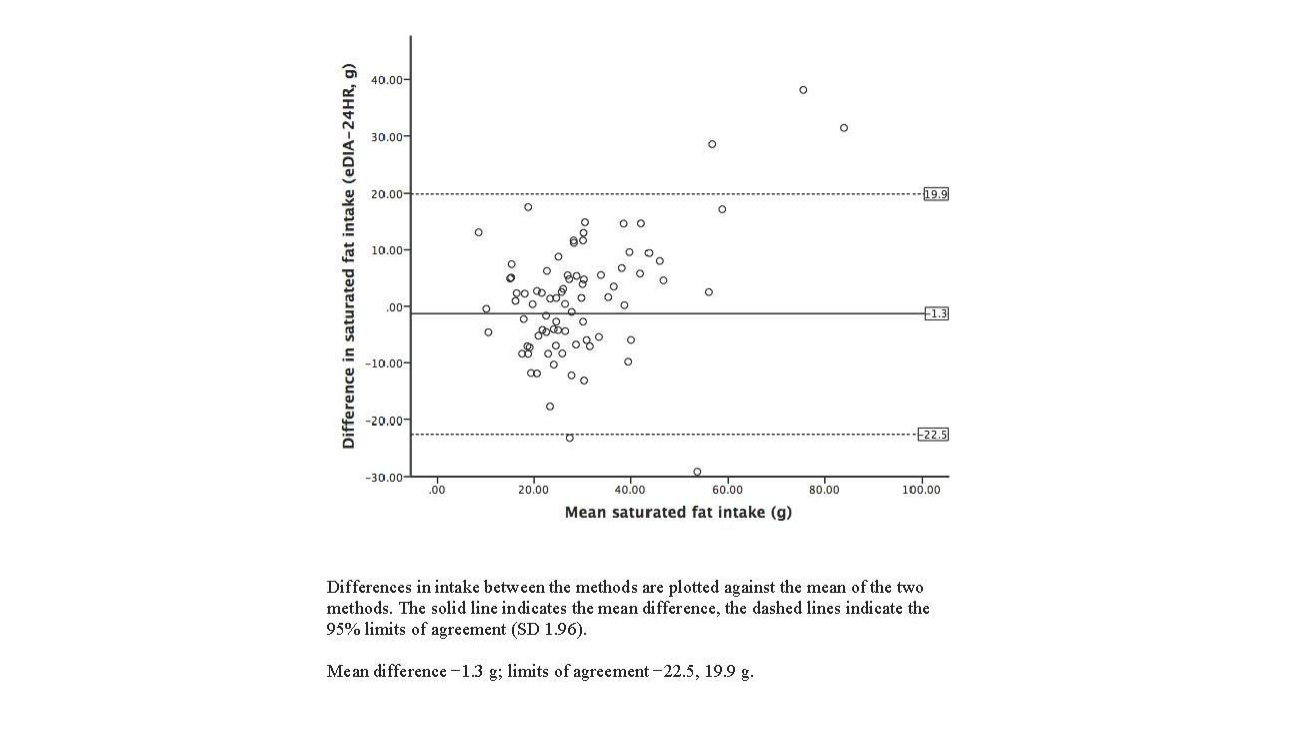
Bland-Altman plot of 24-hour dietary recalls (24HR) and electronic Dietary Intake Assessment (e-DIA) for saturated fat intake.

Bland-Altman plots illustrating the agreement between the 24-hour dietary recalls and e-DIA for energy and selected nutrient intakes are shown in Figures 3-7. For energy intake, the mean difference between e-DIA and 24-hour dietary recall was minimal (−34 kJ) but the 95% limits of agreement were wide (−4062 kJ to 4130 kJ). No systematic bias was detected with random scatter of data points. Similar results were found with other nutrients with small mean differences, with no obvious systematic bias but wide limits of agreement between the two methods.

## Discussion

### Principal Findings

This study is the first to compare the energy and nutrient intakes using a mobile phone food diary app with 24-hour dietary recall as reference measure using an Australian food composition database. Mean intakes of energy and all nutrients were similar in both methods, with no consistently higher or lower values for either method. Correlation coefficients were moderate to strong ranging from 0.55 to 0.78. Cross-classification into quartiles revealed good agreement for energy and all nutrients. In addition Bland-Altman plots showed robust agreement between the e-DIA and 24-hour dietary recalls for energy and all nutrients, without bias and with most data points located within two standard deviations of the mean. The wide limits of agreement suggest that e-DIA is unsuitable to accurately estimate intake at an individual level. However, collectively the results suggest the potential of e-DIA as an assessment tool for dietary analysis at the population level.

These findings are consistent with those of other researchers. Carter et al recently validated a mobile phone app (My Meal Mate) designed to support weight loss [20]. Mean intakes of energy, protein, carbohydrate, and fat were similar using 2-day 24-hour dietary recalls and 7-day electronic food records. Pearson correlations of 0.69 to 0.86 were found for energy and macronutrients, and Bland-Altman analysis of energy intake showed minimal bias but wide limits of agreement between the methods. Comparisons between 24-hour dietary recalls and food records collected using personal digital assistants (PDAs) also produced consistent results with no significant differences between mean intakes of energy, protein, carbohydrate, or fat [21,22]; moderate to strong Pearson correlations (1-day PDA vs 24-hour dietary recalls *r*=0.51-0.80, 7-day PDA vs 24-hour dietary recall *r*=0.72-0.85) [21]; and minimal bias as demonstrated using Bland-Altman plots [21,22].

Mobile phones are also being used for digital imaging to record food and beverage intake [8,9,23-30]. The advantage of these over the digital entry food record is that the respondent burden is considerably reduced with only images recorded and no searching and selection of foods from display lists. With a fiducial marker or reference card, the researcher uses manual or automated methods to assign the food identity and portion size to the image before automatic nutrient analysis. Examples of the use of images with human input into the assignment of foods and quantities include the remote food photography method and the Nutricam dietary assessment method [8,23-26]. Both have been shown to have validity in a free living situation using doubly labelled water to measure energy intake [8,24]. These methods are semiautomated and still require humans to correctly identify foods and amounts. The mobile device food record is an automated system for food identification and volume estimation and offers the recorder the opportunity to see the classifications and correct mislabelled food [9,27-30]. Further development of the process includes increasing correct food recognition and decreasing errors in volume estimation with the automated method. Completely automated systems using digital images provide obvious advantages over digital recording by easing both respondent and researcher burden.

### Limitations and Strengths

Although the use of 24-hour dietary recall was the preferred choice of reference method, it introduces several limitations to the study design. Reliance on memory is a well-documented limitation with participants likely to forget foods consumed the previous day, although the use of the multiple pass method and portion size aids are designed to minimize the impact of errors related to memory. As the 24-hour dietary recall was administered on days that the participants digitally recorded their food records into the e-DIA, there was potential for the recording process to have improved their recall of food and beverages. However, it should be noted that records were deleted from the app at midnight and recalls were conducted up to 22 hours after their deletion. As both methods relied on self-report, more objective measures of dietary intake such as biomarkers are needed to further validate the e-DIA.

Compared with the 2011-2012 Australian Health Survey [31], energy intakes were 8% and 11% lower for males and females, respectively, indicating some degree of under-reporting. This was primarily due to lower reported intakes of carbohydrates (especially sugar) and alcohol in the validation study. University students are a unique population group and are not representative of all young adults, as they are skewed towards higher socioeconomic backgrounds and may have higher digital and computer literacy [32].

The use of the e-DIA also has limitations, including the burden of recording foods prospectively for a prolonged period of time and trouble navigating within the e-DIA tool itself. When entering a food into the e-DIA, participants were presented with a long list of food options that was challenging to navigate. However, the presence of the favorites function relieves some of this burden by prioritizing the food options according to individual preferences. Commercial apps may have shorter lists but this is likely to result in less accurate food records and resulting nutrient intakes.

One of the main strengths of the study is the ability of e-DIA to collect dietary intake data without alerting the participants to their ongoing caloric intake. The app is linked to the Australian national food composition database compiled by Food Standards Australia New Zealand which consists of over 4500 foods [12]. This greatly reduced the need for coding although careful checking of all foods and beverages recorded each day may be useful to obtain reliable nutrient outputs. Another advantage of using the national food composition database is the inclusion of a large range of macronutrients, micronutrients, and other food components. The app is used to record food and beverages consumed in real time and therefore does not rely on memory.

### Conclusions

This validation study demonstrated good agreement between the e-DIA and 24-hour dietary recalls at a group level, and no evidence of bias for energy, macro-, and micronutrients was noted. With the growing popularity of mobile phones among young adults this method of collecting dietary intake is highly acceptable in this population group. Future studies should explore the validity of the e-DIA in larger, more representative samples and employ external biomarkers to reflect usual intakes. Studies assessing the e-DIA’s sensitivity to changes in dietary intake are also required. This would confirm its value as a tool to monitor dietary intake in intervention studies in public health and clinical trials.

## Abbreviations

24HR: 24-hour dietary recall
AUSNUT: Australian Food, Supplement, and Nutrient Database
BMI: body mass index
e-DIA: electronic Dietary Intake Assessment
MUFA: monounsaturated fatty acids
PDA: personal digital assistant
